# Pancreatic ampullary carcinoma with neck metastases: a case report

**DOI:** 10.1186/1757-1626-2-146

**Published:** 2009-10-01

**Authors:** Murat Aksoy, Aziz Sumer, Serkan Sari, Ozgur Mete, Artur Salmaslioglu, Yesim Erbil

**Affiliations:** 1 Department of General Surgery, Faculty of Medicine, Istanbul University, İstanbul, Turkey; 2 Department of General Surgery, Kas State Hospital, Antalya, Turkey; 3 Department of Pathology, Faculty of Medicine, Istanbul University, İstanbul, Turkey; 4 Department of Radiology, Faculty of Medicine, Istanbul University, İstanbul, Turkey

## Abstract

**Background:**

An 18-year-old Turkish woman was referred with a 6-week history of rapidly enlarging cervical mass at the left side.

**Case report:**

She was diagnosed of ampullary carcinoma for which pancreatoduodenectomy was performed 14 months ago. In our patient with a history of malignancy, a rapidly enlarging neck mass was considered a metastasis to the neck. Tumor resection was performed. Histopathological examination revealed the metastasis of the precedent ampullary adenocarcinoma.

**Conclusion:**

Surgery does not improve survival for advanced metastatic ampullary cancer however, it can be mandatory in specific conditions as our patient.

## Introduction

Periampullary neoplasms include carcinomas of the duodenum, ampulla of Vater, distal common bile duct and pancreas. They are considered collectively because of their similar clinical presentation and the difficulty in distinguishing them without examination of a resected specimen [1,2]. More than one half of cases have distant metastasis at diagnosis. Most frequent sites of metastasis are local lymph nodes, lung, liver, adrenal glands, kidney and bones [3-7]. To our knowledge, this is the rare report of a ampullary cancer with neck metastasis.

## Case presantation

An 18-year-old Turkish woman was referred from Oncology Department with a 6-week history of rapidly enlarging cervical mass at the left side of her neck which had been detected by the patient herself. She was diagnosed of ampullary carcinoma for which pancreatoduodenectomy was performed 14 months ago. Histopathological examination revealed well-differentiated adenocarcinoma in the ampullary region. Only one lymph node metastasis was present among 35 dissected lymph nodes. Additionaly, peripancreatic fat tissue invasion was also remarked. She has not a family history of pancreas cancer. Gemcitabine was started 1000 mg/m2 Day 1,8,15/every 28 days for ampullary cancer. After chemotherapy performed, she lost follow-up.

**Figure 1:**
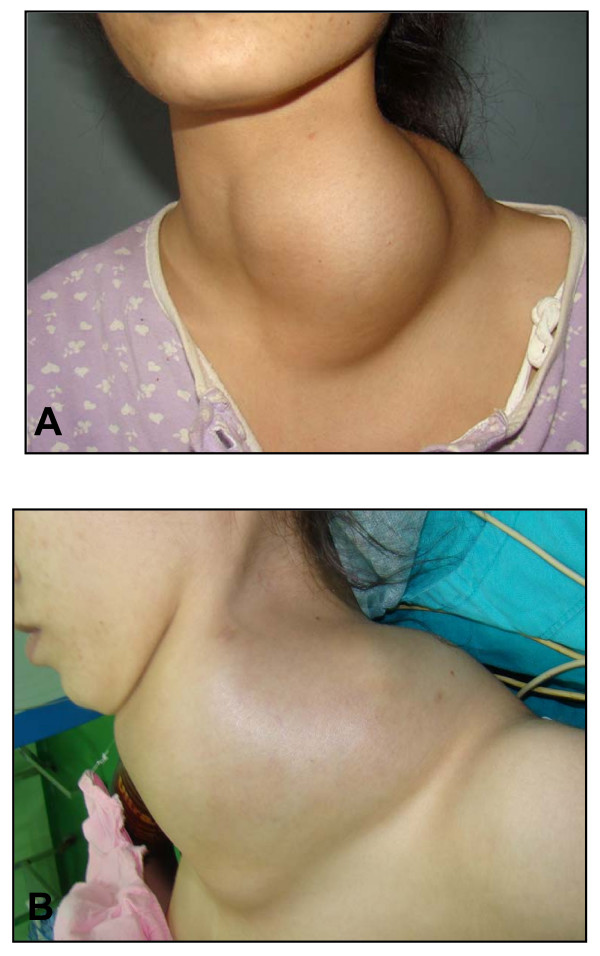
**(A) Huge neck mass before and (B) after biopsy**.

Six months after the chemotherapy, she noticed a rapidly enlarging right neck mass. A 15 cm soft and painless mass involving the left antero-lateral aspect of the neck was palpable. The mass was apparent by inspection (Figure 1A). The mass was fixed to the underlying cervical tissues. There was no known past history of thyroid problems and she was euthyroid. Routine blood chemistry including liver function tests and white blood cell count were normal. Her erythrocyte sedimentation rate and CA-19-9 were 55 mm/h and 2044 U/ml, respectively. Indirect laryngoscopic evaluation revealed normal vocal cords function.

**Figure 2:**
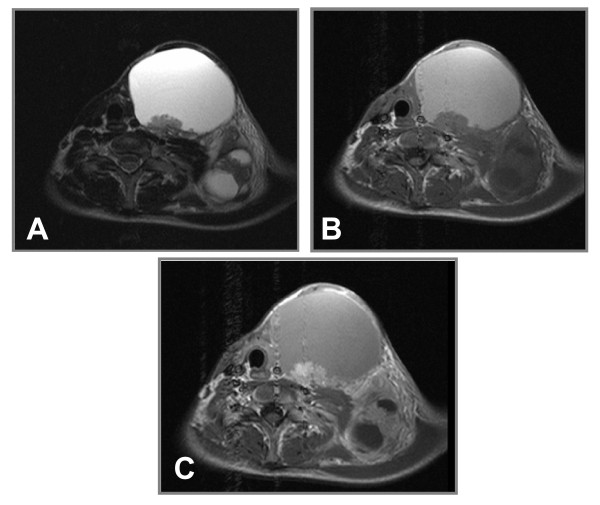
**(A) T2 weighted TSE transverse image demonstrates a large, hyperintense cystic mass with polypoid solid components, displacing middle-line structures, including trachea and esophagus, to the right**. The left common carotid artery is in contact with the medial wall of the lesion. Smaller lesions with similar internal structure can also be detected at the posterosuperior and left posterolateral sides. **(B)**. T1 weighted SE transverse image demonstrates spontaneous hyperintensity in the primary lesion, consistent with intralesional hemorrhage or proteinaceous material. Papillary vegetations are isointense. The smaller posterior components are hypointense with isointense solid vegetations. **(C)**. Contrast enhanced T1 weighted SE transverse image demonstrates heterogeneous enhancement at the papillary components.

Ultrasonography revealed a mixed solid- cystic mass, measuring 13 × 11 cm, with a hyperechogenic capsule. The lesion had a close relationship with the thyroid gland and its borders could not be easily defined. On T2 weighted axial MR images, a hyperintense, dominantly cystic mass with thin borders and papillary vegetations at the posterior wall was occupying the left cervical region. The lesion had displaced the trachea, the thyroid gland and also compressed the left common carotid artery and internal jugular vein. Smaller lesions with similar internal structure could also be detected at the posterosuperior and left posterolateral sides of the primary lesion (Figure 2A). On T1 weighted spin-echo images the main lesion was hyperintense (due to intralesional hemorrhage or presence of proteinaceous material) with isointense papillary components. The smaller lesions were hypointense (Figure 2B). Following contrast material administration, a heterogeneous enhancement could be noted at the papillary components (Figure 2C).

In our patient with a history of malignancy, a rapidly enlarging neck mass was considered a metastasis to the neck. Since imaging studies suggested malignant tumor, a Tru-cut biopsy was performed. Cytologic findings did not provide any further information. After Tru-cut biopsy, severe compressive symptoms and stridor were occured. Those symptoms, accompanied by the enlargement of the mass, were suggestive of intralesional bleeding following biopsy (Figure 1B).

## Surgical technique

**Figure 3:**
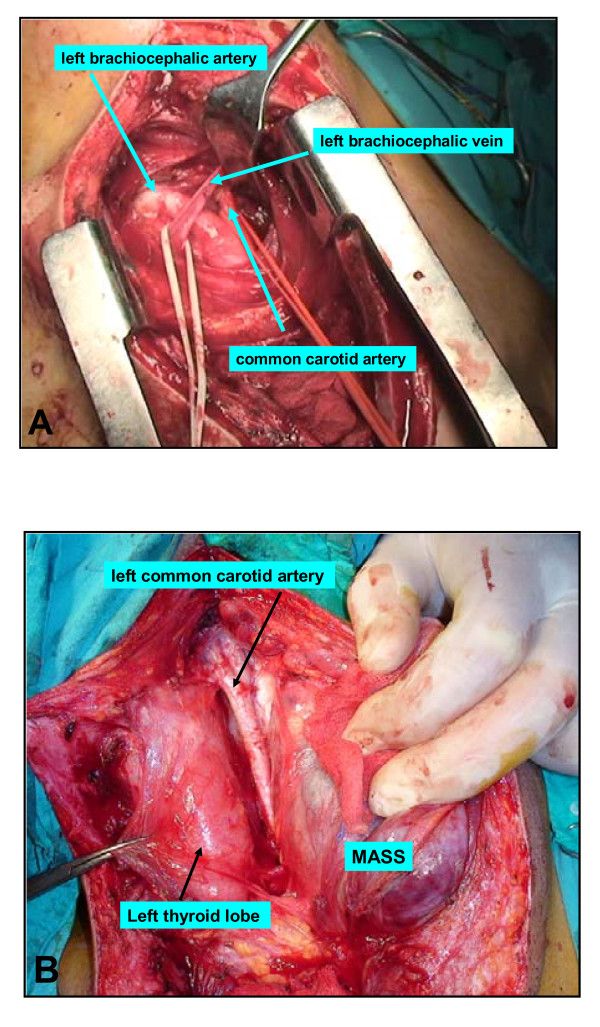
**(A) Isolated left brachiocephalic artery and vein, and left common carotid artery; (B) The appearance of carotid artery, internal jugular vein, left thyroid lobe and cervical mass**.

**Figure 4:**
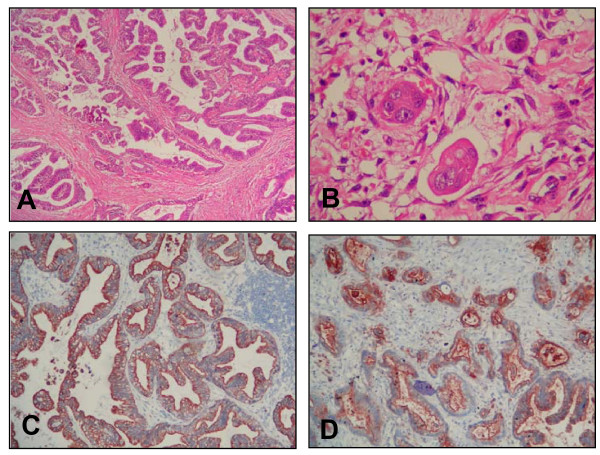
**(A) Metastatic adenocarcinoma forming a mass in the neck soft tissue (H&E, ×20); (B) Lymphovascular tumor thrombus in the surrounding soft tissue (H&E, ×40); (C) Anti-cytokeratin 7 positivity in the tumor cells (Anti-cytokeratin 7, ×20); (D) Anti-CEA positivity in the tumor cells (Anti-CEA, ×40)**.

The patient underwent sternotomy and the left brachiocephalic artery and vein, and left common carotid artery, and left subclavian artery were isolated for vascular control. At the neck exploration, the left cervical mass was voluminous, and adherent to surrounding structures including the carotid artery, internal jugular vein, and left vagal nerve. Left thyroid lobe was found normal in appearance. Left common carotid artery and vagal nerve were liberated from the tumor. Because the tumor invaded left internal jugular vein, it was ligated and divided. After mobilization of the tumor from the adjacent structures, tumor resection was completed (Figure 3A, B). She had an uneventful postoperative course and was discharged home on postoperative day 3. The histopathological examination revealed a metastatic adenocarcinoma containing areas of extensive lymphovascular tumor thrombus (Figure 4A, B). Sections were free of any lymphoid parenchyma and for this reason the mass is regarded as being a metastatic soft tissue mass in the neck region. Anti-cytokeratin 7 and CEA (Carcinoembryogenic antigen) were diffuse and strongly positive in the tumor cells, whereas anti-TTF1 and cytokeratin 20 were negative (Figure 4C, D). Antigenic and morphological features lead us to diagnose the mass as the metastasis of the precedent periampullary adenocarcinoma. At a follow-up visit 3 months after surgery, she remained symptom-free, and a repeat US showed no evidence of recurrence.

## Discussion

Overall, periampullary cancers account for 5% of all gastrointestinal tract malignancies, which can be divided into four groups of tumor entities: periampullary adenocarcinoma originates from the pancreatic duct (pancreatic carcinoma), the mucosa of the ampulla of Vater (ampullary carcinoma), the common distal bile duct segment (distal cholangiocellular carcinoma) or the duodenum (duodenal adenocarcinoma) [1,2].

Panreatic cancer occurs most frequently among the periampullary cancers, accounting for 3% of all gastrointestinal cancers. Carcinoma of the ampulla of Vateri is the second most common periampullary malignancy. Less frequent are distal cholangiocellular carcinoma and duodenal adenocarcinoma of the periampullary region (1). The prognosis of a 5-year survival rate between 1% and 10% for all periampullary cancers still remains a frustrating challenge. However, it has been shown that periampullary tumors, not arising from the pancreatic duct, have a much beter outcome [3].

Morbidity and mortality from pancreatic cancer is conspicuously associated with metastasis; the most frequent sites of metastasis are local lymph nodes, lung, liver, adrenal glands, kidney and bones. Patients with pancreatic cancer have only very rarely been reported to develop metastatic lesions to the brain, skin, and larynx (3-7). Cervical lymph node and brain metastases from pancreatic cancer have rarely been seen and most of them were identified in advanced stage [5,8]. In our case, histopathologic examination did not reveal any lymphoid tissue, which suggests a soft tissue metastasis to the neck. However, it is not possible to prove this hypothesis. Despite the fact that our patient was in an advanced stage, the excision was performed to prevent respiratory difficulties.

While surgery does not improve survival for advanced metastatic ampullary cancer, it can be mandatory in specific conditions as our patient.

## Consent

Written informed consent was obtained from the patient for publication of this case report and accompanying images. A copy of the written consent is available for review by the Editor-in-Chief of this journal.

## Competing interests

The authors declare that they have no competing interests.

## Authors' contributions

MA and YE carried out the operation, AS and YE were contributor in writing the manuscript. SS participated in discussing of the manuscript. OM performed the histological examination of the mass. ARS performed the MRI image examination of the mass. All authors read and approved the final manuscript.
